# Contemplation on wheat vernalization

**DOI:** 10.3389/fpls.2022.1093792

**Published:** 2023-01-06

**Authors:** Zbyněk Milec, Beáta Strejčková, Jan Šafář

**Affiliations:** Institute of Experimental Botany of the Czech Academy of Sciences, Centre of the Region Haná for Biotechnological and Agricultural Research, Olomouc, Czechia

**Keywords:** wheat, vernalization, *VRN*, chromatin methylation, copy number variation, devernalization

## Abstract

Vernalization is a period of low non-freezing temperatures, which provides the competence to flower. This mechanism ensures that plants sown before winter develop reproductive organs in more favourable conditions during spring. Such an evolutionary mechanism has evolved in both monocot and eudicot plants. Studies in monocots, represented by temperate cereals like wheat and barley, have identified and proposed the *VERNALIZATION1* (*VRN1*) gene as a key player in the vernalization response. *VRN1* belongs to MADS-box transcription factors and is expressed in the leaves and the apical meristem, where it subsequently promotes flowering. Despite substantial research advancement in the last two decades, there are still gaps in our understanding of the vernalization mechanism. Here we summarise the present knowledge of wheat vernalization. We discuss *VRN1* allelic variation, review vernalization models, talk *VRN1* copy number variation and devernalization phenomenon. Finally, we suggest possible future directions of the vernalization research in wheat.

## Introduction

Bread wheat (*Triticum aestivum* L.) is an allohexaploid species grown worldwide and adapted to different latitudes and climatic conditions. This ability is related to a variation in the two main genes: *PPD1* (*PHOTOPERIOD1*, photoperiod response) and *VRN1* (*VERNALIZATION1*, vernalization requirement) (Trevaskis et al., 2003; Yan et al., 2003; Beales et al., 2007). *VRN1* belongs to MADS-box (MCM1, AGAMOUS, DEFICIENS, SRF) transcription factors (Yan et al., 2003) and plays a crucial role as an integrator of vernalization-accelerated flowering. Due to the hexaploid nature of bread wheat genome, *VRN1* is present as homoeologs (*VRN-A1*, *VRN-B1* and *VRN-D1*) on chromosomes 5A, 5B and 5D (Snape et al., 2001). Its natural allelic variation is associated with the growth habit - spring or winter (Yan et al., 2004a; Fu et al., 2005). Cold period (=vernalization) accelerates the flowering of winter (autumn-sown) varieties (Chouard, 1960). The length of effective vernalization can range from three to eight weeks (Košner, Pánková 2002; Li et al., 2013). Winter varieties carry recessive *vrn1* alleles. Dominant alleles in the spring varieties are expressed without vernalization and carry mutations in the promoter or the first intron of *VRN1* (Yan et al., 2004a; Fu et al., 2005). The mutations result in the partial or complete inhibition of vernalization requirement. At least one dominant *VRN1* allele confers the spring growth habit (Stelmakh, 1987). In winter wheats, the *VRN1* chromatin undergoes histone methylation changes (H3K4me3 and H3K27me3) during vernalization, possibly affecting the *VRN1* expression (Xiao et al., 2014). Several models of vernalization mechanism have been proposed so far (Amasino, 2004; Yan et al., 2006; Chen and Dubcovsky, 2012; Xiao et al., 2014; Xu et al., 2021; Debernardi et al., 2022). Nevertheless, we still lack a detailed understanding of vernalization molecular mechanism. This review recapitulates current knowledge of the *VRN* alleles and reflects on vernalization models. We also discuss *VRN1* multiple copies and touch on wheat devernalization.

## Vernalization genes

### *VRN1* gene – a central integrator of vernalization-accelerated flowering?

*VRN1* genes have been mapped on the distal end of long arms of 5A (Galiba et al., 1995; Dubcovsky et al., 1998), 5B (Barrett et al., 2002; Iwaki et al., 2002) and 5D (Law et al., 1976). In *T. monococcum*, Yan et al. (2003) cloned the*VRN1* gene from 5A^m^ chromosome and showed *VRN1* expression increased in winter accessions after vernalization in both leaves and apices. Two putative *VRN1* orthologues, *TaVRT-1* and *WAP1*, were identified in bread wheat (Danyluk, 2003; Trevaskis et al., 2003), but later studies reported *TaVRT-1* and *WAP1* were synonyms for the *VRN1* gene (Shitsukawa et al., 2007a; Kane et al., 2007).

Yan et al. (2004b) described *VRN1* allelic variation determined by mutations in the promoter region. The *Vrn-A1a* allele has the highest basal levels of *VRN1* transcripts. It carries the insertion of a mutator DNA transposon called spring foldback element (SFE), which comprises duplication of the partial promoter, complete exon 1 and partial intron 1. The insertion is supposed to disrupt a binding site for a putative *VRN1* repressor. Fu et al. (2005) described large, several-kb-long deletions within the first intron of *VRN1* homoeologs associated with the spring habit. The importance of the *VRN1* gene in the vernalization response and as flower inducer has been generally accepted and supported by many scientific publications (for instance, Pugsley, 1971; Snape et al., 2001; Trevaskis, 2010). An ion-beam-induced mutant (*T. monococcum*) lacking *VRN1* displayed a non-flowering phenotype and was designated *maintained vegetative phase* (*mvp*) (Shitsukawa et al., 2007b). They suggested that *VRN1* was crucial for transitioning from the vegetative to the reproductive stage. A later study (Distelfeld and Dubcovsky, 2010) showed that *mvp* mutants described by Shitsukawa et al. (2007b) were lacking not only *VRN1* but also multiple genes, including *PHYTOCHROME-C* (*PHYC*) and *AGAMOUS-LIKE GENE 1* (*AGLG1*). Chen and Dubcovsky (2012) described *vrn1-*null mutant in tetraploid wheat that was able to flower, responded to vernalization treatment and provided regular seeds. This mutant maintained functional *PHYC* and *AGLG1* genes. Another MADS-box genes, *FRUITFULL2* (*FUL2*) and *FRUITFULL3* (*FUL3*), are the closest *VRN1* paralogs (Preston and Kellogg, 2006). It is likely that some of *PHYC*, *AGLG1*, *FUL2* or *FUL3* might function as redundant flowering genes (Chen and Dubcovsky, 2012).

Natural variations in all three *VRN1* homoeologs of wheat have been reported (**Table 1**). All identified mutations have been designated as individual alleles, but not all were experimentally confirmed to affect the heading time. The fact that dominant *VRN1* alleles carry indels compared to recessive (intact) alleles may suggest they are evolutionary younger.

**Table 1:** The list of *VRN1* alleles reported in hexaploid (6x), tetraploid (4x) and diploid (2x) wheat.

Allele	First reported in	Reference
VRN1		
***vrn-A1***	6x	(Yan et al., 2004a)
***Vrn-A1a, Vrn-A1a.1***	6x	(Yan et al., 2004a)
***Vrn-A1a.2***	6x	(Muterko et al., 2016)
***Vrn-A1a.3***	4x	(Yan et al., 2004a)
***Vrn-A1b***	6x	(Yan et al., 2004a; Strejčková et al., 2021)
***Vrn-A1b.2- Vrn-A1b.6***	4x, 6x	(Muterko et al., 2016)
***Vrn-A1c***	6x	(Yan et al., 2004a; Fu et al., 2005)
***Vrn-A1d***	4x	(Yan et al., 2004a)
***Vrn-A1e***	4x	(Yan et al., 2004a)
***Vrn-A1f***	4x, 6x	(Golovnina et al., 2010)
***VRN-A1f-like***	4x	(Ivaničová et al., 2016)
***vrn-A1f-del***	4x	(Shcherban et al., 2016)
***Vrn-A1f-del/ins***	4x	(Shcherban et al., 2016)
***Vrn-A1f-ins***	4x	(Shcherban et al., 2016)
***Vrn-A1g***	2x, 4x	(Golovnina et al., 2010)
***Vrn-A1h***	2x	(Golovnina et al., 2010)
***Vrn-A1i***	4x	(Muterko et al., 2016)
***VRN-A1AUS28709 Ai2***	6x	(Steinfort et al., 2017)
***Vrn1h/VRN-A1ins***	2x	(Dubcovsky et al., 2006; Shcherban et al., 2015)
***Vrn-A1k***	4x	(Muterko and Salina, 2017)
***Vrn-A1L***	4x	(Fu et al., 2005)
***vrn-A1u***	4x	(Golovnina et al., 2010)
***vrn-A1u´***	4x	(Shcherban et al., 2015)
***vrn-B1***	6x	(Yan et al., 2004a)
***Vrn-B1a*** Vrn-B1a*	6x 4x	(Fu et al., 2005) (Golovnina et al., 2010)
***Vrn-B1b***	6x	(Santra et al., 2009)
***Vrn-B1c*** Vrn-B1c**	6x	(Chu et al., 2011; Milec et al., 2012; Shcherban et al., 2012)
Vrn-B1d**	6x	(Zhang et al., 2018)
***Vrn-B1f***	6x	(Strejčková et al., 2021)
***Vrn-B1ins***	4x	(Chu et al., 2011)
***vrn-D1***	6x	(Yan et al., 2004a)
***Vrn-D1a***	6x	(Fu et al., 2005)
***Vrn-D1b***	6x	(Zhang et al., 2012)
***Vrn-D1c***	6x	(Zhang et al., 2015)
***Vrn-D1s***	6x	(Muterko et al., 2015)
***vrn-D1r***	6x	(Strejčková et al., 2021; Makhoul et al., 2022)
***Vrn-D4***	6x; Special case	(Kippes et al., 2015)
Vrn-D1x***	6x	(Makhoul et al., 2022)
VRN2		
***VRN-A2***	4x	(Dubcovsky and Dvorak, 2007)
***VRN-B2***	4x	(Dubcovsky and Dvorak, 2007)
***VRN-B2a-1***	6x	(Tan and Yan, 2016)
***VRN-B2a-2***	6x	(Tan and Yan, 2016)
***VRN-D2***	2x	(Distelfeld et al., 2009b)
FT1 (VRN3)		
***Vrn-A3b-h1, CS VRN-A3 allele, TAFTAh1, FT-A1 haplotype H1***	6x	(Bonnin et al., 2008; Chen et al., 2020; Nishimura et al., 2021)
***Vrn-A3a-h1, TN26 VRN- A3 allele***	4x	(Nishimura et al., 2018; Nishimura et al., 2021)
***Vrn-A3b-h2, TN28 VRN-A3 allele***	4x	(Nishimura et al., 2018; Nishimura et al., 2021)
***Vrn-A3a-h2***	4x, 6x	(Nishimura et al., 2021)
***Vrn-A3a-h3***	4x	(Nishimura et al., 2021)
***Vrn-A3a-h4***	4x	(Nishimura et al., 2021)
***Vrn-A3a- h5***	4x	(Nishimura et al., 2021)
***Vrn-A3a-h6***	4x	(Nishimura et al., 2021)
***Vrn-A3c-h1***	4x, 6x	(Nishimura et al., 2021)
***Vrn-A3c-h2***	4x	(Nishimura et al., 2021)
***TAFTAh2, FT-A1* haplotype H2**	6x	(Bonnin et al., 2008; Chen et al., 2020)
***TAFTAh3, FT-A1* haplotype H3**	6x	(Bonnin et al., 2008; Chen et al., 2020)
***TAFTAh4, FT-A1* haplotype H4**	6x	(Bonnin et al., 2008; Chen et al., 2020)
***vrn-B3***	6x	(Yan et al., 2006)
***Vrn-B3a***	6x	(Yan et al., 2006)
***Vrn-B3b***	6x	(Chen et al., 2013)
***Vrn-B3c***	6x	(Chen et al., 2013)
***Vrn-B3d***	6x	(Berezhnaya et al., 2021)
***Vrn-B3e***	6x	(Berezhnaya et al., 2021)
***TaFTBBT21***	6x	(Bonnin et al., 2008)
***TAFTDh1***	6x	(Bonnin et al., 2008)
***TAFTDh2***	6x	(Bonnin et al., 2008)

*Santra et al. (2009) described a novel dominant allele *Vrn-B1b* in hexaploid wheat variety Alpowa. Following the nomenclature, they referred to the dominant *Vrn-B1* allele reported by Fu et al. (2005) as *Vrn-B1a*. This *Vrn-B1a* allele carries nearly 7-kb deletion within the first intron. In tetraploid wheat, Golovnina et al. (2010) reported the *Vrn-B1a* allele with a 127-bp insertion in the promoter; they did not sequence the whole *Vrn-B1* gene body.

**Chu et al. (2011) first reported the *Vrn-B1c* allele. Later, Milec et al. (2012) and Shcherban et al. (2012) independently reported the same new *Vrn-B1* allele and incorrectly designated it as *Vrn-B1c*. This allele was renamed to *Vrn-B1d* in the Catalogue of gene symbols for wheat: 2013-2014 supplement https://shigen.nig.ac.jp/wheat/komugi/genes/macgene/supplement2013.pdf. Therefore, the *Vrn-B1d* allele reported by Zhang et al. (2018) should be referred to as *Vrn-B1e*.

***Makhoul et al. (2022) reported the same 17-bp deletion in the first intron of *VRN-D1* as Strejčková et al. (2021) and designated this allele *vrn-D1r*.

### *VRN2* gene – long-day flowering repressor

**Figure 1:**
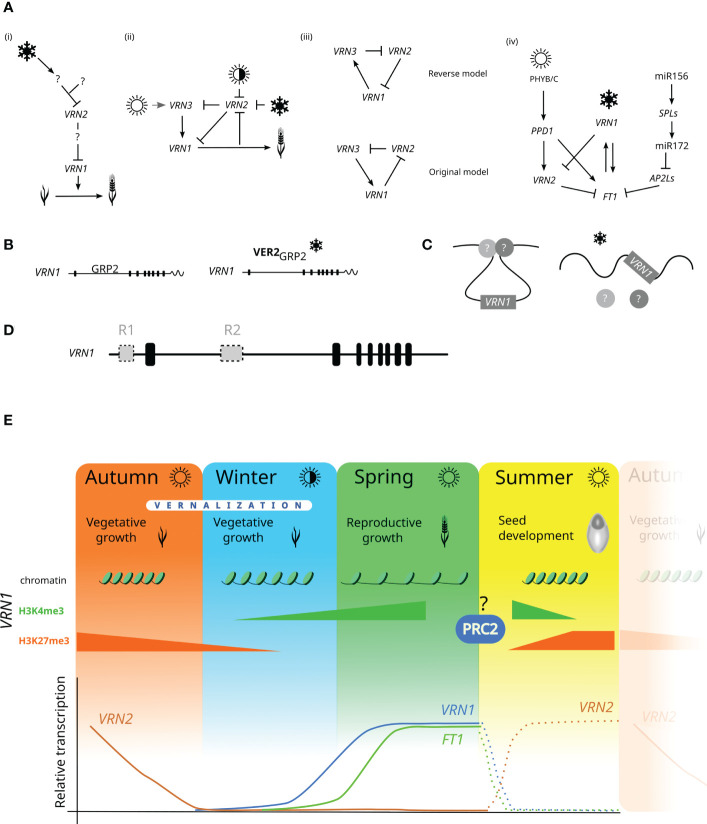
Vernalization mechanism working models. **(A)** Development of working models for *VRN1, VRN2* and *VRN3* (=*FT1*) interactions: **(i)** (Amasino, 2004), **(ii)** (Yan et al., 2006), **(iii)** (Chen and Dubcovsky, 2012), (iv) (Debernardi et al., 2022) **(B)** working model for possible posttranscriptional mechanism of vernalization requirement, (Xiao et al., 2014) **(C)** working model for *VRN1* transcription induced by non-coding RNA (Xu et al., 2021). Arrows indicate gene upregulation; lines ending in the crossed bar indicate gene repression. The snowflake symbol represents vernalization, and white sun symbol represents the long day, and the half-white/half-black sun symbol represents the short day. A question mark indicates an unknown element. *FT1* = *VRN3*. **(D)** The hypothetical model of two putative repressors (R1, R2) acting independently in either the *VRN1* promoter or the first intron. **(E)** *VRN1* chromatin state, histone methylation levels, and *VRN* expression profiles in the winter wheat life cycle. Dashed lines represent the predicted progress of *VRN* transcriptions levels.

*VRN2* codes for a zinc finger motif protein and includes two duplicated *ZCCT* genes (Yan et al., 2004b). The CCT domain was first described in *Arabidopsis* proteins CONSTANS, CONSTANS-like and TIMING OF CAB1 (Strayer et al., 2000). Wheat homoeologs *VRN-A2*, *VRN-B2* and *VRN-D2* were mapped on chromosomes 5A, 4B and 4D, respectively (Yan et al., 2004b; Tan and Yan, 2016). In autumn-sown winter wheat, the flowering induction is repressed by the *VRN2* gene during long days as the *PPD1* promotes *VRN2* transcription (Dubcovsky et al., 2006; Shaw et al., 2020). *VRN2* represses a flowering promoter, *FLOWERING LOCUS T* (*FT1* = *VRN3*). Cold and short days during winter downregulate *VRN2*, releasing both *VRN1* and *FT1* transcription (Yan et al., 2004b; Dubcovsky et al., 2006) (**Figure 1Aiv**).

### *FT1* (=*VRN*3) gene – flowering promoter

The vernalization-related gene located on chromosome 7B of the spring wheat substitution line Hope was reported (Law, 1966; Law and Wolfe, 1966) and designated *VRN5* (Stelmakh, 1987; Law and Worland, 1997). Later, the name was changed to *VRN-B4*, corresponding to its chromosome localisation (McIntosh et al., 1998). Yan et al. (2006) showed that *VRN-B4* is the *VRN3* gene orthologous to the *FT* gene in *Arabidopsis*. *FT* encodes for the mobile protein that moves in the leaves and the apical meristem (Corbesier et al., 2007). The FT protein/mRNA is more likely the long sought flowering compound called florigen (reviewed in (Turck et al., 2008)). In wheat, high levels of *VRN1* after vernalization induce *FT1* transcription (Distelfeld et al., 2009a) (**Figure 1Aiv**).

## Models of vernalization mechanism

Vernalization has been known and studied for a long time, but the precise molecular mechanism still waits to be revealed. A growing number of studies kept bringing new findings that were used in developing several models of how vernalization may work (Amasino, 2004; Yan et al., 2006; Chen and Dubcovsky, 2012; Xiao et al., 2014; Debernardi et al., 2022). The models are depicted in **Figure 1**. The first simplified model (Amasino, 2004) shows the vernalization pathway with *VRN2* repressing *VRN1* and several other unknown genes (**Figure 1Ai**). The vernalization was suggested to repress *VRN2* expression. The *VRN2* expression was also downregulated by a short day (during autumn), while *VRN2* repressed both *VRN1* and *FT1* (**Figure 1Aii**). Shimada et al. (2009) developed transgenic plants overexpressing *FT1*, causing *VRN1* upregulation and *VRN2* downregulation. *FT1* is highly transcribed even in both Δ*vrn1*-null and Δ*vrn1*-*vrn2*-null mutants lacking functional *VRN1* and *VRN2* genes (Chen and Dubcovsky, 2012). They proposed the model where *VRN1* represses *VRN2*, which downregulates *FT1*, and *VRN1* and *FT1* mutually upregulate one another, creating a positive feedback loop. The reworked vernalization model was now referred to as “original”, and Shimada’s model as “reverse” (Chen and Dubcovsky, 2012) (**Figure 1Aiii**). The most recent working model presented by Debernardi et al. (2022) supports findings reported by (Chen and Dubcovsky, 2012), showing *VRN1* downregulation of *VRN2*. During long days in the autumn, *PPD1* upregulates *VRN2*, which downregulates *FT1*, preventing wheat from flowering (**Figure 1Aiv**). Debernardi et al. (2022) also identified a conserved pathway integrating plant age into flowering regulation. This pathway involves the expression of miR172 acting like a flowering promotor but its targets *APETALA2-like1* and *5* (*AP2L1*, *AP2L5*) function as flowering repressors.

These models describe the interaction of *VRN1*, *VRN2* and *FT1*. However, they do not explain the molecular mechanism at the DNA level. Dominant *Vrn1* alleles have higher basal transcription levels, minimising the vernalization requirement. Indels present in the dominant alleles may have removed or disrupted a putative binding site for an unknown *VRN1* repressor. Xiao et al. (2014) described a mechanism of *VRN1* induction during vernalization. The proposed model suggests glycine-rich RNA-binding protein (GRP2) as a repressor preventing *VRN1* transcript accumulation. Before vernalization, GRP2 directly binds to the *VRN1* pre-mRNA in the region characterised as critical. It comprises the *VRN1* intron approximately from 1.3 kb to 4.2 kb (from the start codon) (Fu et al., 2005). The cold treatment induces the expression of *VER2* and increases the GRP2 *O*-GlcNAcylation level. *VER2* creates a complex with GRP2 that releases *VRN1* transcript accumulation and induces flowering promotion (**Figure 1B**). Xiao et al. (2014) also reported histone methylations might participate in the vernalization response. During the cold period, the H3K27me3 levels at *VRN1* chromatin decrease while levels of H3K4me3 increase. The high levels of H3K4me3 are associated with active gene transcription, while increased H3K27me3 levels are linked with gene repression (Roh et al., 2006; Wysocka et al., 2006; Zhang et al., 2007). These changes are targeted to the first half of the *VRN1* first intron, which is in concordance with the previous model (Alonso-Peral et al., 2011) and findings reported in barley (Oliver et al., 2009). It also supports the significance of the *VRN1* critical region in vernalization response. Xiao et al. (2014) developed wheat transgenic lines with *GRP2* overexpression (*GRP-OE*) or *GRP2* silencing by RNA interference (RNAi). The results showed that the mean heading time of *GRP-OE* lines did not significantly differ from the wild type (winter variety JH9). The *GRP2-*RNAi lines had reduced mean heading time (155 days) compared to the wild type (165 days). These lines were always earlier than wild type, irrespective of the length of the vernalization treatment. Although the difference was statistically significant, it did not approximate the spring varieties’ mean heading time. Depending on the growth conditions, the spring wheat heading time can range from 25 to 90 days (Li et al., 2017; Huang et al., 2018). Thus, we can hypothesise about the presence of another putative, more powerful *VRN1* repressor. One could inspire from the study in *Brachypodium distachyon* where the *REPRESSOR OF VERNALIZATION* (*RVR1*) was described (Woods et al., 2017). They showed that mutation in *RVR1* bromo-adjacent homology and transcriptional elongation factor S-II domains leads to reduced H3K27me3 levels of *VRN1* chromatin and results in accelerated flowering without vernalization.

The histone methylation of *VRN1* chromatin observed during vernalization (Xiao et al., 2014) might result from Polycomb repressive complex 2 (PRC2) activity. This complex is a histone methyltransferase consisting of four subunits (Bantignies and Cavalli, 2011). The SET domain in the catalytic subunit Enhancer of zeste [E(z)] is associated with the H3K27 trimethylation. The vernalization requirement is reset in the next sexual generation, probably during seed development (reviewed in (Trevaskis, 2010) (**Figure 1E**). The genes coding for individual PRC2 subunits in bread wheat have been recently reported (Strejčková et al., 2020), but the role of PRC2 in wheat vernalization still has to be unravelled. Lomax et al. (2018) characterised *Brachypodium* mutant flowering rapidly under non-vernalizing conditions. A single nucleotide polymorphism (SNP) in the *ENHANCER OF ZESTE-LIKE 1* (*EZL1*) was associated with global reduction of H3K27me3, which corresponds with *EZL1* function in the PRC2 activity.

Flowering can be also regulated by long non-coding RNAs (lnc RNAs). In *Arabidopsis*, lnc RNAs derived from both strands of *FLOWERING LOCUS C* (*FLC*) affect *FLC* transcription (Helliwell et al., 2011; Heo and Sung, 2011; Kim and Sung, 2017; Kim et al., 2017). The more recent model in bread wheat describes *VRN1* regulation by non-coding RNA transcribed from the *VRN1* sense strand (Xu et al., 2021). This alternative transcript (*TaVRN1* alternative splicing, *VAS*) is induced during the first weeks of vernalization and includes the first exon and the first intron. In non-vernalised winter wheat, *VRN1* forms a loop due to the activity of unknown proteins. *VAS* stimulates the production of *VRN1* transcripts by engaging other proteins, such as TaRF2a and TaRF2b. The loop is released during vernalization, which leads to the complete transcription of *VRN1* (**Figure 1C**). *VAS* includes the short alternative transcript reported previously (Xiao et al., 2014).

## *VRN* copy number variation

Chromosomal segments are subject to deletions or duplications. Such rearrangements larger than 1 kb are called copy number variation (CNV) (Żmieńko et al., 2014). CNVs played a significant role in human evolution but are also an important factor causing diseases, including cancer (reviewed in (Hastings et al., 2009)). In polyploids like bread wheat, CNV refers to the number of gene copies per haploid genome (Würschum et al., 2015). *VRN-A1*, *VRN-B1* and *VRN-D1* homoeologs are located on different chromosomes (Snape et al., 2001), but individual *VRN1* genes can also be present in multiple copies on the same chromosome. CNV has been reported mainly for the *VRN-A1* gene, which can be present from one to two copies (dominant *Vrn-A1a*) or up to four copies (recessive *vrn-A1*) (Díaz et al., 2012; Würschum et al., 2015; Strejčková et al., 2021). Two copies of *VRN-B1* were observed in the hexaploid species *T. compactum* and *T. spelta* (Muterko and Salina, 2019). No CNV for *VRN-D1* have been described so far (Strejčková et al., 2021), but screening more varieties may reveal multiple *VRN-D1* copies. Although the word “copy” implies identical sequences of repeated sections, several types of *VRN1* copy number variation exist. Actually, this fact helped to identify individual copies. Using current sequencing and assembling techniques, it would be extremely hard to distinguish one copy from another if they were 100% identical. The first type of CNV is the SNP in *VRN-A1* exons 4 and 7, reported in wheat accessions carrying two or more copies (Díaz et al., 2012; Muterko and Salina, 2018). An advanced case of CNV displays the *Vrn-A1c* allele present in the spring Afghanistan land race IL369. The *vrn-A1c* allele has two copies: one recessive (intact) copy and one dominant copy with the deletion in the first intron (Fu et al., 2005; Díaz et al., 2012). The *VRN-D4* gene originated by translocation of ∼ the 290-kb region from the distal part of the long arm of the 5A chromosome to the proximal region of the short arm of chromosome 5D (Kippes et al., 2015). This region included the *vrn-A1* gene; therefore, *VRN-D4* might be considered an unusual case of CNV as it involves translocation between two haploid subgenomes. The CNV can have a diverse effect on flowering: an extra *vrn-A1* copy translocated from 5A to 5D chromosome (= *VRN-D4* gene), also carrying SNP (A367C), resulted in the spring growth habit with no vernalization requirement (Kippes et al., 2015). The *Vrn-A1c* allele confers spring growth habit due to the mutated copy carrying the large deletion within the first *VRN1* intron. The higher number of *vrn-A1* copies within the same 5A chromosome, associated with SNPs in *VRN1* exons 4 and 7, led to an increased vernalization requirement; plants with more than one copy needed a prolonged cold period to start *VRN-A1* transcription (Díaz et al., 2012; Li et al., 2013). Recently, a speed vernalization (SV) method was reported (Cha et al., 2022). They showed that the SV effectivity varied among varieties with the different numbers of *vrn-A1* copies. Wheat variety Charger (three *vrn-A1* copies) flowered quicker when speed-vernalized for four weeks, while variety Claire (one *vrn-A1* copy) had a shorter flowering time under two or six weeks of speed vernalization.

Regarding *VRN2*, Tan and Yan (2016) reported duplication of *the VRN-B2* gene in hexaploid wheat, but no significant effect on flowering time was observed.

No increased number of *FT1* was described in hexaploid wheat so far. In barley (*Hordeum vulgare* L.), four *HvFT1* copies significantly accelerated flowering time (Nitcher et al., 2013). Wheat and barley are evolutionary close to each other, suggesting a possible, unrevealed existence of multiple *FT1* copies in wheat.

## Devernalization

Vernalization results in the change from the vegetative to the reproductive growth. The initial metabolic changes lead to morphological changes when the shoot apical meristem begins to produce floral primordia instead of leaf primordia (Yong et al., 2003). The effect of vernalization treatment can be partially or completely removed (in some species) by several days of heat treatment called devernalization. The most effective temperature was considered 30 – 40°C (Bernier, 1981). The heat treatment (around five days) needed to be applied directly after the end of vernalization; it becomes ineffective after a few days of plant growth at ordinary temperatures (Michaels, Amasino 2000). In bread wheat, several studies were performed to determine if vernalization-induced developmental changes could be also reversed (Gregory and Purvis, 1948; Chujo, 1970; Xiu-zhen et al., 1987; Yong et al., 2003; Xu et al., 2019). During devernalization experiments, vernalised wheat plants were exposed to higher temperatures, ranging from 18°C to 35°C. The treatment was associated with delayed flowering, changes gene expression patterns, or protein content changes. The length of the cold treatment reported in the studies varied from 21 to 40 days, which might not be enough to complete vernalization. The vernalization requirement duration can be genotype-dependent (Li et al., 2013). Its genetic nature is not fully understood, but might be linked with *VRN-A1* locus or mutations in acetylglucosamine transferase *TaOGT1* (Díaz et al., 2012; Li et al., 2013; Kippes et al., 2015; Fan et al., 2021). Study in *Arabidopsis* demonstrated that the effect of six-week-vernalization might be erased by heat treatment (30°C). It was associated with the elimination of epigenetic mark H3K27me3 from *FLC* (Bouché et al., 2015). This H3K27me3 removal caused *FLC* reactivation, which led to delayed flowering. Thus, the devernalization phenomenon in cereals might be putative only and needs further research.

## Conclusion and future perspectives

The knowledge of wheat vernalization mechanism has expanded enormously in the last 20 years once *VRN* genes have been cloned and characterised. The advances in molecular methods enabled us to identify sizeable allelic variability in the *VRN1* gene and to update vernalization models. Despite all these achievements, the main question remains: what is the actual molecular mechanism of wheat vernalization? In bread wheat, the *VRN1* allelic variation data suggests two different evolutionary events resulting in the spring growth habit – the deletion within the *VRN1* first intron and the insertion of the mutator DNA transposon in the *VRN1* promoter region. Both deletion and insertion may disturb the binding site for the putative *VRN1* repressor(s). The *Vrn-A1a* allele has the first intron intact (same with recessive allele) but carries the insertion in the promoter region. This allele has the highest basal *VRN1* expression level associated with shorter heading time than the dominant *Vrn-B1* and *Vrn-D1* alleles. However, vernalization increases *Vrn-A1a* transcription, supporting the previously described role of the first intron. As mentioned previously, the *Vrn-A1a* allele has duplicated promoter and exon 1. This might be the reason for such high transcript levels, but the function of this duplication remains undiscovered. The current knowledge about the wheat vernalization mechanism supports the hypothesis that there might be two independent putative *VRN1* repressors: one targeting the *VRN1* promoter region and the other interacting with the first intron (Alonso-Peral et al., 2011) (**Figure 1D**). Besides, the role of multiple *vrn-A1* copies in the vernalization response should be studied in more detail. The position of individual copies within the genome is not known and there is no information whether they have the same expression pattern. Finally, our understanding of how vernalization requirement is reset in the next generation is limited. It possibly occurs during the seed development and may involve PRC2-related histone modification of *VRN1* chromatin.

We should not forget to mention an integral part of vernalization: how plants sense the duration of cold period. Longer cold treatment increases *VRN1* transcript levels suggesting a quantitative character of vernalization (reviewed in (Trevaskis, 2010)). The length of sufficient vernalization varies among winter wheats. Several hypotheses regarding *VRN1* were suggested but they were not in concordance: copy number variation, amino acid change or SNP in the putative repressor binding site (Díaz et al., 2012; Li et al., 2013; Xiao et al., 2014). Another explanation was proposed in *Arabidopsis* (Kyung et al., 2022). The long-term cold-mediated response might employ circadian clock regulators CIRCADIAN CLOCK ASSOCIATED 1 and LATEELONGATED HYPOCOTYL.

Recent technical and methodological advances will further help to untangle vernalization. Increased availability of the genome and transcriptome sequencing, along with the improvements in computational biology, may reveal new molecular mechanisms involved in the vernalization pathway. VanGessel et al. (2022) recently demonstrated transcriptional signatures of inflorescent development in the tetraploid wheat variety Kronos. The gene expression atlas of the floral meristem based on single nucleus RNA-seq data was developed latterly in barley (Neumann et al., 2022). Adapting the new techniques of targeted mutagenesis could help to develop the new alleles for functional studies.

There might be more unknown genes and their interactions participating in wheat vernalization. We could compare this phenomenon to mosaic assembling; once all fragments are in their place, we will see the complete picture.

## Author contributions

ZM proposed the manuscript. ZM, BS and JS participated in the research and provided original results. All authors contributed to the article and approved the submitted version.
